# The right tool for the right question: contrasting biogeographic patterns in the notothenioid fish *Harpagifer* spp. along the Magellan Province

**DOI:** 10.1098/rspb.2021.2738

**Published:** 2022-04-13

**Authors:** N. I. Segovia, C. A. González-Wevar, J. Naretto, S. Rosenfeld, P. Brickle, M. Hüne, V. Bernal, P. A. Haye, E. Poulin

**Affiliations:** ^1^ Departamento de Ciencias Ecológicas, Instituto Milenio de Ecología y Biodiversidad (IEB), Universidad de Chile. Las Palmeras 3425, Ñuñoa, Santiago, Chile; ^2^ Departamento de Biología Marina, Facultad de Ciencias del Mar, Universidad Católica del Norte, Larrondo 1281, Coquimbo, Chile; ^3^ Instituto Milenio en Socio-ecología Costera (SECOS), Coquimbo, Chile; ^4^ Instituto Milenio Biodiversidad de Ecosistemas Antárticos y subAntárticos (MI-BASE), Valdivia, Chile; ^5^ Instituto de Ciencias Marinas y Limnológicas (ICML), Facultad de Ciencias, Universidad Austral de Chile, Casilla 567, Valdivia, Chile; ^6^ Centro de Investigación en Dinámicas de Ecosistemas de Altas Latitudes (Fondap IDEAL), Universidad Austral de Chile; ^7^ Costa Humboldt, Puerto Varas, Los Lagos, Chile; ^8^ Laboratorio de Ecosistemas Antárticos y sub-Antárticos, Universidad de Magallanes, Chile; ^9^ South Atlantic Environmental Research Institute (SAERI), PO Box 609, Stanley Cottage, Port Stanley, Falkland Islands, UK; ^10^ Centro de Investigación para la Conservación de los Ecosistemas Australes (ICEA), Punta Arenas, Chile

**Keywords:** Southern South America, phylogeography, population genomics, sub-Antarctic fishes, next-generation sequencing

## Abstract

Molecular-based analysis has become a fundamental tool to understand the role of Quaternary glacial episodes. In the Magellan Province in southern South America, ice covering during the last glacial maximum (20 ka) radically altered the landscape/seascape, speciation rates and distribution of species. For the notothenioid fishes of the genus *Harpagifer,* in the area are described two nominal species. Nevertheless, this genus recently colonized South America from Antarctica, providing a short time for speciation processes. Combining DNA sequences and genotyping-by-sequencing SNPs, we evaluated the role of Quaternary glaciations over the patterns of genetic structure in *Harpagifer* across its distribution in the Magellan Province. DNA sequences showed low phylogeographic structure, with shared and dominant haplotypes between nominal species, suggesting a single evolutionary unit. SNPs identified contrastingly two groups in Patagonia and a third well-differentiated group in the Falkland/Malvinas Islands with limited and asymmetric gene flow. Linking the information of different markers allowed us to infer the relevance of postglacial colonization mediated by the general oceanographic circulation patterns. Contrasting rough- and fine-scale genetic patterns highlights the relevance of combined methodologies for species delimitation, which, depending on the question to be addressed, allows discrimination among phylogeographic structure, discarding incipient speciation, and contemporary spatial differentiation processes.

## 1. Introduction

Biogeographical boundaries, identified as the coincidence of species distribution limits, generally reflect abiotic discontinuities acting directly on the survival of taxa, but they can also reflect both evolutionary consequences and historical climate changes. Quaternary glacial cycles are considered main drivers of current distribution patterns in cold-temperate and polar near-shore biotas, as continental ice sheet expansions during glacial maxima led to the eradication of most marine benthic organisms in large ice-covered areas [1–5]. Thus, Quaternary ice ages, particularly the last glacial maximum (LGM) around 20 ka, radically altered the demography and the geographical range of higher latitude species and populations. Glacial ice sheet advances and retreats also modelled the distribution of intraspecific genetic variation and patterns of population structure [2,4,6,7]. A vast array of records from the Northern Hemisphere provided the empirical basis for the expansion–contraction (E–C) model of Pleistocene biogeography [8], a fundamental paradigm for Quaternary biogeographers. This model describes the response of populations and species to climate change [2,9,10] and helps to understand how cold-temperate taxa survived the LGM at lower-latitude refugia and then recolonized higher latitudes through range expansion following the deglaciation process [9,11].

During the LGM, the Pacific Magellan margin was almost fully covered by the Patagonian Ice Sheet, expanding over 480 000 km^2^ with a volume of around 500 000 km^3^ [12–15]. Radical glacial landscape/seascape shifts across this area resulted in the periodic temporal elimination of the associated terrestrial and near-shore marine biota [4,16–18].

Ice sheet retreats during warmer periods allowed the colonization of new vacant habitats creating opportunities for isolation and speciation [2,3,19,20]. Quaternary glacial cycles led to regional isolation and extinction, shaping the current patterns of species diversity in cold-temperate areas of southern South America [4,21].

Zoogeographic delimitations of the Southern Ocean provinces have considered the Magellan Province as a key sub-Antarctic area that includes the southern tip of South America and the Falkland/Malvinas Islands [22–24], and the new *Biogeographic Atlas of the Southern Ocean* [25] recognized the Magellan Province as a single sub-Antarctic province clearly separated from other sub-Antarctic ones [24]. During the last two decades, mtDNA-based phylogeographic studies across the Magellan Province showed that terrestrial and marine biota underwent demographic dynamics associated with the E–C model, with recent population expansions following the LGM. Such patterns have been recorded in vertebrates [26–28] and plants [29,30].

Several studies conducted in near-shore marine species were restricted to the Pacific margin of South America several taxa [31–35], including fishes [36,37]. These studies support a strong impact of the last LGM on species restricted to shallow marine habitats in areas heavily impacted by continental ice sheet advances. Although several near-shore marine species are distributed across the entire Magellan Province, few phylogeographic studies have been conducted across the region [34,38,39]. Phylogeographic patterns detected when including the Falkland/Malvinas Islands ranged from the absence of genetic differentiation [39,40] to marked phylogeographic differentiation [19,33,34] and the presence of clearly divergent species-level clades [41,42].

The Southern Ocean notothenioid fishes originated in Antarctica and have dominated in diversity, abundance and biomass since the local extinction of most of the ichthyofauna during the Eocene [43]. The monogeneric notothenioid family Harpagiferidae includes littoral benthic species currently distributed in the Southern Ocean. Two nominal species are currently recognized in the Magellan Province: *Harpagifer bispinis* (Forster 1801), endemic to the southern tip of South America, and *H. palliolatus* Richardson 1845, endemic to the Falkland/Malvinas Islands. However, Richardson [44], in the original description of *H. palliolatus*, was sceptical if the morphological differences were enough to diagnose them as different species: 'I have seen only one example of this form of Harpagifer and am not convinced on its being specifically distinct from bispinis, notwithstanding the very different way in which it is colored' [44]. Accordingly, the specific status conferred to *H. palliolatus* from the Falkland/Malvinas is still doubtful and requires revision.

So far, one study has been performed on the genus *Harpagifer* through mtDNA comparisons between South American *H. bispinis* and the Antarctic species *H. antarcticus* [37]. Phylogenetic reconstructions supported the presence of different species of *Harpagifer* on the two sides of the Drake Passage, albeit with low levels of genetic divergence. Divergence time estimates suggest separation during the Pleistocene, between 1.2 and 0.8 Ma [37], much more recently than the divergence between South American and Antarctic congeneric species of other marine species [5,45–47], including other notothenioid species [20,48,49]. Hüne *et al*. [37] proposed a scenario of a northward movement of the Antarctic Polar Front during the Great Patagonian Glaciation of the Quaternary (0.9–1 Ma). This northward shift would have allowed the sub-Antarctic colonization of *Harpagifer* from the Antarctic Peninsula towards South America through the Scotia Arc. This scenario of recent sub-Antarctic colonization leads to the question of whether if it is possible that speciation took place in South America (*H. bispinis* versus *H. palliolatus*) in such a short evolutionary time, posing the possibility that the Magellan Province harbours a single species.

During recent decades, molecular-based analyses using traditional markers (mtDNA and nucDNA sequences) have become the main tool to understand and unravel the role of Quaternary glacial episodes on the distribution and demography of populations, generally improving the understanding of biodiversity and systematics through species delimitation methods [2,6,8,40,50]. Accordingly, the sudden increasing availability of genome-based data in non-model organisms has significantly improved the spatial resolution of genetic structure and potentially allows inferences concerning the historical and contemporary diversification of organisms [51]. Also, there is a trend to use anonymous genome-wide markers obtained through reduced representation sequencing (RRS) to address systematic and taxonomic questions [52–55]. However, such data do not generally allow one to distinguish population genetic structure from divergence process as they provide allelic frequency comparisons [56–58]. In this study, we used the combined information of DNA sequences and non-targeted SNPs to shed light about the role of Quaternary glacial events in the genetic structure and the potential role of post-glacial colonization processes in the contemporary patterns of gene flow of the genus *Harpagifer* in South America. Using this genus in southern South America as a study model, we aim to unravel the role and potential of each kind of molecular marker to address specific questions about population genetic structure, phylogeography and species delimitation.

## 2. Material and methods

**Figure 1.**
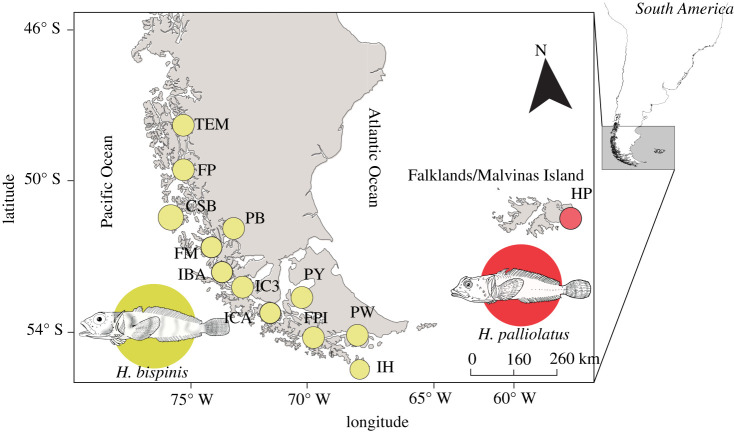
Sampling sites for the nominal species *Harpagifer bispinis* and *H. palliolatus* across Pacific Patagonia and the Falkland/Malvinas Islands. (Online version in colour.)

Populations of *Harpagifer bispinis* were collected from the intertidal of 12 localities along the Pacific Patagonia between 48.73° S, 74.05° W and 55.84° S, 67.37° W, and *H. palliolatus* specimens were collected at Hookers Point (51.03° S, 57.7° W) in the Falkland/Malvinas Islands (figure 1; electronic supplementary material) [59]. All specimens were preserved in 95% ethanol. DNA extractions were done using the DNeasy Blood and Tissue Kit (Qiagen, USA). The quantity and integrity of DNA were measured using both Nanodrop 2 (Thermo, USA) and Qubit 4 (Thermo, USA).

Partial fragments of two mitochondrial (D-loop and COI) and one nuclear gene (Rhodopsin) were amplified through PCR (electronic supplementary material, table S2). Amplicons were purified and sequenced in both directions at Macrogen Inc. (Seoul, South Korea). Alignments were obtained with Geneious R10 (https://www.geneious.com). The haplotype phases of the rhodopsin sequences were inferred using Phase 2.1 in DnaSP 6.0 [60].

For RRS, samples were sequenced through a genotyping-by-sequencing (GBS) method at the Biotechnology Center in the University of Wisconsin using, after optimization, the *ApeKI* restriction enzyme. After enzyme digestion, each DNA fragment was linked to a barcode adaptor to recognize it *in silico* and libraries were prepared using a HiSeq2000 (Illumina, USA) platform. Reads were visualized in FastQC 0.10.1 for quality checks. SNP-calling was carried out with the pipeline Universal Network-Enabled Analysis Kit (UNEAK) in Tassel v. 3 [61]. We used a minor allele frequency of 0.05 and a site minimum call rate of 0.75 to ensure that at least 75% of the individuals in each SNP were covered for at least 1 tag. After filtering, we estimated Hardy–Weinberg equilibrium deviations per locus and per population with Arlequin 3.5.2.2 [62] using 10 000 permutations. *p*-values were corrected with a false discovery rate (FDR) correction (*q*-value = 0.05), and SNPs that appeared in HW disequilibrium in at least 60% of the populations were removed from the dataset.

We estimated levels of polymorphism in *H. bispinis* and *H. palliolatus* populations for the COI, D-loop and rhodopsin datasets in DNasP 6.0 [60] using standard diversity indices: haplotype number, number of polymorphic sites, haplotype diversity, the average number of pairwise differences, and nucleotide diversity. Genealogical relationships were reconstructed using median-joining haplotype networks in PopART (http://popart.otago.ac.nz). Pairwise distances (*p-distances*) were calculated using Kimura-2-parameter.

Patterns of population structure were determined through pairwise *F*_ST_ and *Φ*_ST_ in Arlequin v. 3.5 [62] and their significance using 10 000 permutations. We evaluated phylogeographic structure using Permut [63] by comparing the F_ST_ and *Φ*_ST_ values using 10 000 random permutations of genetic distance among haplotypes. Finally, we performed the Bayesian clustering algorithm implemented in Geneland v. 3.1.4 [64].

SNPs putatively under diversifying selection were identified using an F_ST_ *outlier* approach implemented in Bayescan 2.1 [65]. Considering that such loci tend to be highly differentiated and exacerbate the genetic structure, they were not considered for analyses. A total of five separate runs were performed with 500 000 iterations and a 10% burn-in period to assure the convergence of the MCMC and a prior odd of 1000. A FDR correction of *q*-values of 0.05 was applied in Bayescan to avoid the occurrence of false positives.

Expected and observed heterozygosity and allele richness with rarefied allele counts across the study area were calculated using Genodive v. 3.05 [66], and private allelic richness and diagnostic alleles among genetic groups were calculated in HP-Rare 1.0 [67].

Pairwise F_ST_ analyses were calculated in Arlequin v. 3.5 [62] with the significance tested through 10 000 permutations of individuals between localities. With Structure 2.3.4 [68], we evaluated the probability of assignment of a given individual to a genetic cluster using 10 replicate runs performed in parallel using Strauto [69] with 500 000 MCMC and 10% burn-in. Optimal *K* values were estimated using Evanno's method [70], using delta *K*. Discriminant analysis of principal components (DAPC) in the R package adegenet [71] was used to identify genetic clusters with the information about the geographical origin of each individual. The optimal number of clusters for DAPC was estimated with the k-means clustering with Bayesian information criterion (BIC) in the function *find.clusters* using 100 000 iterations, 100 PC and six discriminant functions.

Additionally, we tested for population structure using spatial location and geographic distance between individuals using the R package conStruct to dissociate the population structure from continuous clines of genetic variation [72]. With conStruct, we estimated the effect of both isolation by distance and discrete population structure based on individuals' relationships [72]. We run five independent chains with three layers, with 100 000 iterations. The contribution of each layer was calculated using cross-validation runs.

Finally, contemporary asymmetric gene flow patterns between each determined cluster were estimated with BayesAss 3.04 [73]. With BayesAss, we used the results of the previous clustering analyses and identified those individuals that putatively migrate from another genetic group using the number of times that each individual assigns to the other populations/genetic group. The rates of contemporary immigration among clusters were estimated using 10 000 iterations and a burn-in period of 10%.

## 3. Results

We obtained 126 COI sequences of 669 nucleotide positions with no stop codon and no indels, corresponding to 99 and 27 individuals of *H. bispinis* and *H. palliolatus*, respectively. Alignment included 36 haplotypes and 31 variable positions (4.63%) of which 13 were parsimony informative (41.93%). D-loop data included 34 haplotypes in 177 sequences, corresponding to 135 and 42 individuals of *H. bispinis* and *H. palliolatus,* respectively. Rhodopsin alignment consisted of 6 haplotypes in 86 sequences after Phase, corresponding to 24 and 19 individuals of *H. bispinis* and *H. palliolatus,* respectively. Levels of genetic diversity in nominal species were generally moderate to low (table 1). For instance, haplotype diversity for COI and D-loop in *H. bispinis* ranged from 0.121 to 0.789 (table 1). Similar values for COI were recorded for *H. palliolatus* (*h* = 0.718), with lower genetic diversity for D-loop in this nominal species (*h* = 0.220) (table 1). Rhodopsin was the least diverse marker, with only six haplotypes in total and low overall haplotype diversity for both nominal species. *H. palliolatus* exhibited higher diversity for rhodopsin than *H. bispinis* (h: *H. bispinis* = 0.121, *H. palliolatus* = 0.572) (table 1).

**Table 1.** Genetic diversity for COI, D-loop and rhodopsin data for *H. bispinis* and *H. palliolatus*. The table shows sampling size (N), polymorphic sites (S), number of haplotypes (H), haplotype diversity (h), the average number of differences between pairs of sequences (∏) and nucleotide diversity (π).

nominal species	N	S	H	h	∏	π
*H. bispinis*	99/135/48	25/ 27/3	29/33/3	0.789/0.743/0.121	1.42/1.69/0.20	0.00213/0.0037/0.0003
*H. palliolatus*	27/42/38	10/3/4	9/3/5	0.718/0.220/0.572	1.18/0.27/1.12	0.00176/0.0006/0.0014
total	126/177/86	31/29/5	36/34/6	0.783/0.652/0.358	1.42/1.39/0.67	0.00212/0.003/0.00084

Molecular distances (Kimura 2-parameter) between the nominal species were low and varied between 0.099% (rhodopsin) and 0.221% (D-loop). Global *Φ*_ST_ comparisons showed low but significant structure for the three studied markers considering both nominal species (*Φ*_ST_: COI = 0.03 [*p* = 0.02], D-loop = 0.12 [*p* < 0.001], rhodopsin = 0.19 [*p* < 0.001]) (table 2). Using pairwise values of *Φ*_ST,_ several locations from Patagonia appeared as non-significantly differentiated from Falkland/Malvinas Islands after FDR correction (electronic supplementary material, Information). Analyses using PERMUT and GENELAND for each of the three markers did not discriminate between significant grouping or evidence of phylogeographic signal (table 2).

**Table 2.** Summary of genetic and geographic structure analysis of COI, D-loop and rhodopsin for *Harpagifer* using three approximations: *F*_ST_ in Arlequin, *F*_ST_ > *Φ*_ST_ t in Permut and spatial clustering using Geneland (optimal *k* value).

	Arlequin	Permut	Geneland
	*F*_ST_ (*p*)	*F*_ST_ > *Φ*_ST_ (*p*)	*k*
COI	0.0326 (0.026)	0.035–0.091 (0.0058)	1
D-loop	0.1166 (<0.001)	0.152–0.089 (0.9344)	1
Rho	0.1924 (<0.001)	0.178–0.163 (0.6)	1

**Figure 2.**
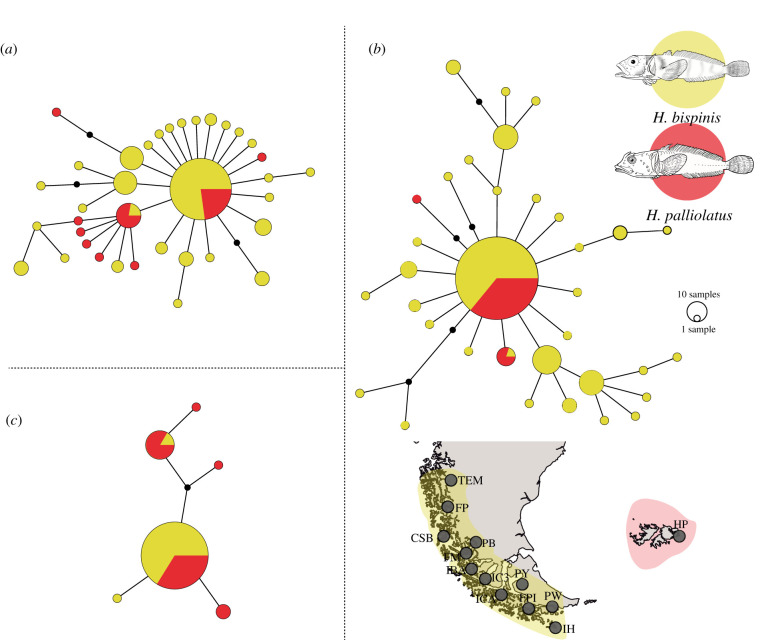
Haplotype network and sampling map for DNA sequences of the nominal species *H. bispinis* and *H. palliolatus* along the Magellan Province. This figure shows the lineage relationship for (*a*) COI, (*b*) D-loop and (*c*) rhodopsin. Inset shows the sampling map for both nominal species along Pacific Patagonia and the Falkland/Malvinas Islands. (Online version in colour.)

Genealogical reconstructions of haplotypes using mtDNA markers (COI and D-loop) showed a star-like pattern, with a single broadly distributed dominant haplotype (figure 2*a*,*b*). *H. bispinis* and *H. palliolatus* shared several haplotypes, including the dominant ones, and showed low frequencies of private haplotypes. For the nuclear marker rhodopsin, derived haplotypes were linked by no more than three mutational steps to the dominant and broadly distributed haplotype (figure 2*c*).

We obtained a total of 3061 SNPs for *Harpagifer* in South America. Bayescan determined that 68 SNPs showed strong or very strong evidence of being putatively under diversifying selection and in consequence were removed from the dataset. Finally, 2993 putatively neutral non-targeted SNPs were used to evaluate the spatial genetic structure and contemporary gene flow of Patagonia and Falkland/Malvinas in *Harpagifer* populations.

**Figure 3.**
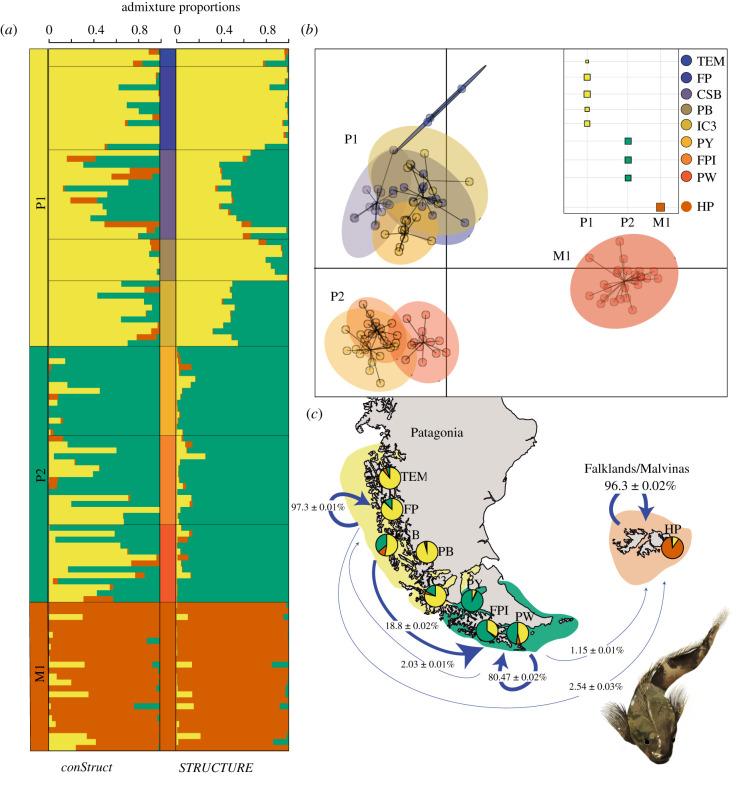
Spatial genetic structure in *Harpagifer* in Patagonia and Falkland/Malvinas Islands. (*a*) Shows the results of a spatial model in conStruct (left panel) and Structure (right panel) results with optimal *k* = 3, (*b*) DAPC scatter plot showing the first two axes for the optimal separation (*k* = 3) determined using BIC and proportion of individuals that belong to each cluster. (*c*) Gene flow patterns estimated with BayesAss considering the three clusters along Patagonia–Malvinas. Arrow directions and their thickness represent asymmetrical migration and proportion of individuals found in each cluster with a high probability of being migrants and standard deviation, respectively. Arrows within clusters represent the proportion of self-recruitment estimated per cluster. Values below 1% are not shown. Additionally, in each site is plotted the pies of the average admixture proportions for each of the three layers (P1, P2 and M1) modelled with the spatial model of conStruct using allele frequencies, geographical location and geographical distance among individuals. Illustration of *Harpagifer bispinis* by Daniela Silva. (Online version in colour.)

In contrast with results with DNA sequence data, there was a significant geographic structure found in the study area (figure 3). With similar levels of genetic diversity (table 3), this structure consistently showed three groups: two groups in Patagonia, P1 (From TEM to IC3) and P2 (From PY to PW), whose limit was coincident with the Strait of Magellan, and a third and highly differentiated group (M1) in the Falkland/Malvinas (Hooker Point) (figure 3*a*,*b*). Independently of whether analyses were based on individuals (Structure), a spatial model incorporating isolation by distance (conStruct) or sampled localities (DAPC), they showed the same pattern. With clustering approaches, Falkland/Malvinas appears as an isolated group with slight signals of admixture with Patagonia with conStruct, Structure and BayesAss (figure 3*a*–*c*). For Structure, and in agreement with DAPC, three main groups were detected as optimal clustering using Evanno's method: two in Patagonia (P1 and P2) and one in Falkland/Malvinas. Using HPRare, no private alleles were detected in Falkland/Malvinas, while 58 were detected in Patagonia. Furthermore, no diagnostic allele was found comparing Patagonia with Falkland/Malvinas.

**Table 3.** Genetic Diversity for SNP-GBS of *Harpagifer*. This table shows the acronyms for each location (same as DNA sequences), number of alleles corrected after rarefaction (Ar), expected (He) and observed heterozygosity (Ho) and inbreeding coefficient (Gis).

acron	N	Ar	Ho	He	Gis
TEM	3	1.477	0.376 ± 0.26	0.478 ± 0.13	0.267
FP	13	1.612	0.196 ± 0.14	0.310 ± 0.14	0.361
CSB	13	1.676	0.256 ± 0.16	0.310 ± 0.14	0.188
PB	13	1.574	0.260 ± 0.19	0.358 ± 0.34	0.287
IC3	12	1.621	0.215 ± 0.18	0.337 ± 0.14	0.318
PY	19	1.729	0.306 ± 0.18	0.313 ± 0.14	0.013
FPI	14	1.714	0.276 ± 0.17	0.319 ± 0.14	0.121
PW	14	1.688	0.238 ± 0.17	0.324 ± 0.13	0.223
HP	27	1.647	0.256 ± 0.17	0.298 ± 0.15	0.124

Contemporary gene flow determined with BayesAss (figure 3*c*) showed asymmetrical migration rates between the two groups in Patagonia following a northern migration pattern, with a migration rate of 18.8% from P1 to P2. A lower migration rate of 2.03% was recorded from P2 to P1 (figure 3*c*). The same analysis showed limited and asymmetrical migration rates from Patagonia to Falkland/Malvinas Islands, with an estimated proportion of 3.69% migrants in total from both groups in Patagonia (P1 and P2) (figure 3*c*), with 96.3% of the individuals of Falkland/Malvinas with ancestry in the same island (self-recruitment), results that agreed with admixture proportion pies estimated with conStruct (figure 3*c*).

## 4. Discussion

The biogeography of the Southern Ocean largely reflects the historical and contemporary interaction among plate tectonics, oceanography, climate and the biota through the Cenozoic, and particularly during the last 50 Ma [74–76]. Our results showed that traditional DNA markers did not detect evidence of discrete evolutionary units between putative South American species of *Harpagifer*, which exhibited very low (and even absence of) genetic differentiation. Neither of the clustering analyses using traditional DNA sequences was able to recognize significant groups associated with the nominal species *H. bispinis* and *H. palliolatus*. In fact, each of the analysed markers consistently showed the presence of shared dominant haplotypes in both nominal species. Moreover, levels of genetic distance between *H. bispinis* and *H. palliolatus* (0.213 in D-loop) are lower than those recorded for intraspecific population studies of fishes (0.343 ± 0.05%) [77]. Hence, *Harpagifer* populations across the Magellan Province represent a single evolutionary unit, supporting a recent biogeographic ichthyological revision of sub-Antarctic fish fauna suggesting a single Magellan ecoregion [78]. Finally, following the taxonomic priority principle, we suggest that a single species, *Harpagifer bispinis* (Forster 1801), is currently distributed along the Pacific and the Atlantic margins of South America and in the Falkland/Malvinas Islands.

Low levels of phylogeographic structure in the study area have been reported in the pulmonates *Siphonaria fuegiensis* [34] and *S. lessonii* [39]. Also, *Harpagifer* populations in South America exhibited middle to low levels of mtDNA and nuclear DNA genetic diversity compared to other Magellan fishes including the notothenioid *Eleginops maclovinus* [79] and the galaxiid *Galaxias maculatus* [36], being most comparable to patterns of genetic diversity recorded in Antarctic fish species including *Lepidonotothen* spp. [80,81] and *Trematomus* [82]. Low levels of genetic diversity and structure recorded in *Harpagifer* may be explained by the Quaternary glacial history of the study area, as well as the ecology of the species. The colonization of areas that were formerly glaciated would have involved a series of genetic bottlenecks and therefore recolonized areas should exhibit low genetic diversity dominated by few haplotypes and high frequency of sequences from the founding population. Accordingly, the main phylogeographic patterns in *Harpagifer* provide evidence for the E–C model of Quaternary biogeography. Alternatively, the absence of genetic structure found in *Harpagifer* using DNA sequences could also be a consequence of the recent diversification estimated for the genus that may have colonized the Magellan Province less than approximately 1 Ma [37].

Complementing the results obtained with traditional DNA sequences, SNP-GBS identified a contrasting pattern; three genetic groups in *H. bispinis*, two of them restricted to Pacific Patagonia, one located north of the Strait of Magellan (P1) another south of it (P2), as well as a third differentiated group from the Falkland/Malvinas Islands (M1). Our analyses showed a marked separation between Pacific Patagonia and the Falkland/Malvinas Islands, a pattern that could be explained mainly by the geographic distance between populations. Despite these areas being highly differentiated, we found evidence of low but significant asymmetrical gene flow where at least 3% of the individuals collected in the Falkland/Malvinas Islands are likely to be derived from Pacific Patagonia. In the opposite direction, a negligible percentage (less than 0.1%) of the individuals from Patagonia could have been from the Falkland/Malvinas Islands. Contemporary asymmetrical gene flow from Pacific Patagonia towards the Falkland/Malvinas Islands in *H. bispinis* is expected under the general oceanographic circulation pattern in this region and has been also found in patellogastropods [19,33] and pulmonates [39] using traditional sequence markers. Limited (but significant) contemporary gene flow could explain the absence of phylogeographic structure detected through traditional DNA analyses, preventing population divergence between the areas.

An interesting spatial pattern that emerged from the SNP-based structuring analyses in *H. bispinis* is the presence of two groups in Pacific Patagonia separated by the Strait of Magellan. After Quaternary glacial cycles, the Strait of Magellan is mainly a long waterway that separates Patagonia from Tierra del Fuego, with a minimum width of 2 km and a maximum depth of approximately 1800 m. Although this area has been considered as a transition zone by some authors [83,84], to date there is no molecular evidence supporting the presence of different populations at both sides of this geographical feature. Possible explanations for this discontinuity may be associated with the ecology of the species, its reproductive behaviour [85] and the larval ecology, which may decrease the connectivity across geographically complex areas such as that found in the Patagonian fjords. Due to the absence of swim bladder and their negative buoyancy, adults of *H. bispinis* exhibit major restrictions of movement. Hence, individuals are mainly benthic inhabitants of coastal rocky shores and intertidal pools, and have the lowest natant ability among sub-Antarctic nototheniids [86]. Accordingly, active dispersion of individuals should occur only during the free-living pelagic larval period, which may last approximately three months in conspecifics (*H. antarcticus* [87]). However, *Harpagifer* larvae tend to be retained close to the coast [85,88], limiting the effective dispersion capability and in consequence the connectivity between populations [87]. The Strait of Magellan seems to act as the main connectivity barrier for a benthic organism with limited effective larval dispersion. According to our migration estimations, and as expected under the general circulation pattern and the direction of the main Cape Horn current, there is an asymmetric and poleward gene flow pattern from (P1) north to south (P2), a pattern previously reported in the same area for the limpets *Nacella magallanica* [19] and *N. mytilina* [33].

Our results in *Harpagifer bispinis* represent the first evidence of a genetic discontinuity across this area and show the importance of the use of fine-scale molecular markers in genetic differentiation studies, which may play a key role in the knowledge of the Quaternary evolution of near-shore benthic fauna. A similar example occurs in the 30° S transition zone in the Humboldt Current System [89]. In the ascidian *Pyura chilensis*, this well-known phylogeographic break was unrecognized by traditional markers [90] but detected with SNPs using spatial genetic structure analysis, further suggesting that the contemporary influence of this break is due to environmental differences north/south of the zone and consequent local adaptation processes [91,92].

Since this is the first study in a Magellan near-shore marine benthic species performed with SNPs, further phylogeographic studies using fast-evolving markers and oceanographic biophysical models of the circulation patterns in this complex area are necessary to corroborate and support these findings, and to assess if this isolation by distance pattern could be maintained by local adaptation processes.

## 5. Conclusion

Since non-targeted SNPs are increasingly used for phylogenetic inferences and species delimitation analyses [52–55,93], we point out here the need to complement such an approach with traditional DNA sequences. In our study, in the absence of the information provided by mtDNA and nucDNA sequences, and due to the strong spatial structure between Patagonia and Falkland/Malvinas detected through SNPs, we would have probably supported the hypothesis of two *Harpagifer* species in southern South America or at least supported evidence of incipient speciation. Nevertheless, such a conclusion would have been a consequence of intraspecific spatial structure rather than historical genealogical patterns associated with historical divergence. Using different species concepts (e.g. phylogenetic, biological and genealogical), our data confirm that *Harpafiger* in South America does not include two separate evolutionary units as Richardson [44] hypothesized in the original description of *H. palliolatus*. Based on the low levels of pairwise distances recorded among *Harpagifer* populations across Pacific Patagonia and the Falkland/Malvinas Islands, our results do not support a scenario of incipient speciation or divergence. Furthermore, the absence of diagnostic alleles between Patagonia and Falkland/Malvinas provides strong evidence that the detected patterns with SNPs in South American populations of *Harpagifer* are a consequence of contemporary patterns of genetic structure and gene flow. This pinpoints the necessity to verify the phylogenetic status of each evolutionary unit using different approaches before drawing genealogical conclusions based solely on non-targeted SNPs. In the Southern Ocean, traditional DNA molecular markers have been extensively used for species delimitation analyses (see [40,41,94,95]). Recently, RRS data has also been successfully applied as a tool for phylogenetic inference [50,96,97], but in all those cases, SNP data were complemented with available molecular, morphological or taxonomic evidence. Our study therefore supports the idea that depending on the original question to be addressed, what we are detecting using traditional markers and SNP data is (i) potential phylogeographic structure that could show a divergence process and incipient speciation (discarded in our case), and (ii) differentiation processes linked to drift-migration equilibrium models. Sukumaran & Knowles [56,57] suggest that multi-species coalescent approaches for species delimitation regularly delimit population structure rather than actual cladogenetic processes. Contrasting results between traditional and fast-evolving markers will help to put in perspective the reliability of SNPs from RRS techniques (e.g. GBS and RADseq) approaches in the absence of a reference genome in species delimitation, phylogenetic inferences and as a genealogical approach in evolutionary biology.

Finally, as previously demonstrated in shallow marine benthic organisms including fishes [98], invertebrates [3,34,40,42,99] and bacteria [100], the taxonomy of the Southern Ocean biota requires major revisions, including molecular and morphological analyses.

## Data Availability

Individual genotypes for SNP-GBS data are available from the Dryad Digital Repository: https://doi.org/10.5061/dryad.jwstqjq9q [101]. DNA sequences for haplotypes are available from GenBank for d-loop (accession nos. OL347639–OL347672), COI (accession nos. OL339430–OL339465) and Rhodopsin (accession nos. OL347673–OL347678). The data are provided in the electronic supplementary material [102].
